# Microglia in retinal angiogenesis and diabetic retinopathy

**DOI:** 10.1007/s10456-024-09911-1

**Published:** 2024-04-02

**Authors:** Aiyan Hu, Mirko H. H. Schmidt, Nora Heinig

**Affiliations:** Institute of Anatomy, Medical Faculty Carl Gustav Carus, Technische Universität Dresden School of Medicine, Fetscherstr 74, 01307 Dresden, Germany

**Keywords:** Angiogenesis, Diabetic retinopathy, Microglia modulation, Microglia state, Retinal neovascularization

## Abstract

Diabetic retinopathy has a high probability of causing visual impairment or blindness throughout the disease progression and is characterized by the growth of new blood vessels in the retina at an advanced, proliferative stage. Microglia are a resident immune population in the central nervous system, known to play a crucial role in regulating retinal angiogenesis in both physiological and pathological conditions, including diabetic retinopathy. Physiologically, they are located close to blood vessels and are essential for forming new blood vessels (neovascularization). In diabetic retinopathy, microglia become widely activated, showing a distinct polarization phenotype that leads to their accumulation around neovascular tufts. These activated microglia induce pathogenic angiogenesis through the secretion of various angiogenic factors and by regulating the status of endothelial cells. Interestingly, some subtypes of microglia simultaneously promote the regression of neovascularization tufts and normal angiogenesis in neovascularization lesions. Modulating the state of microglial activation to ameliorate neovascularization thus appears as a promising potential therapeutic approach for managing diabetic retinopathy.

**Graphical abstract:**

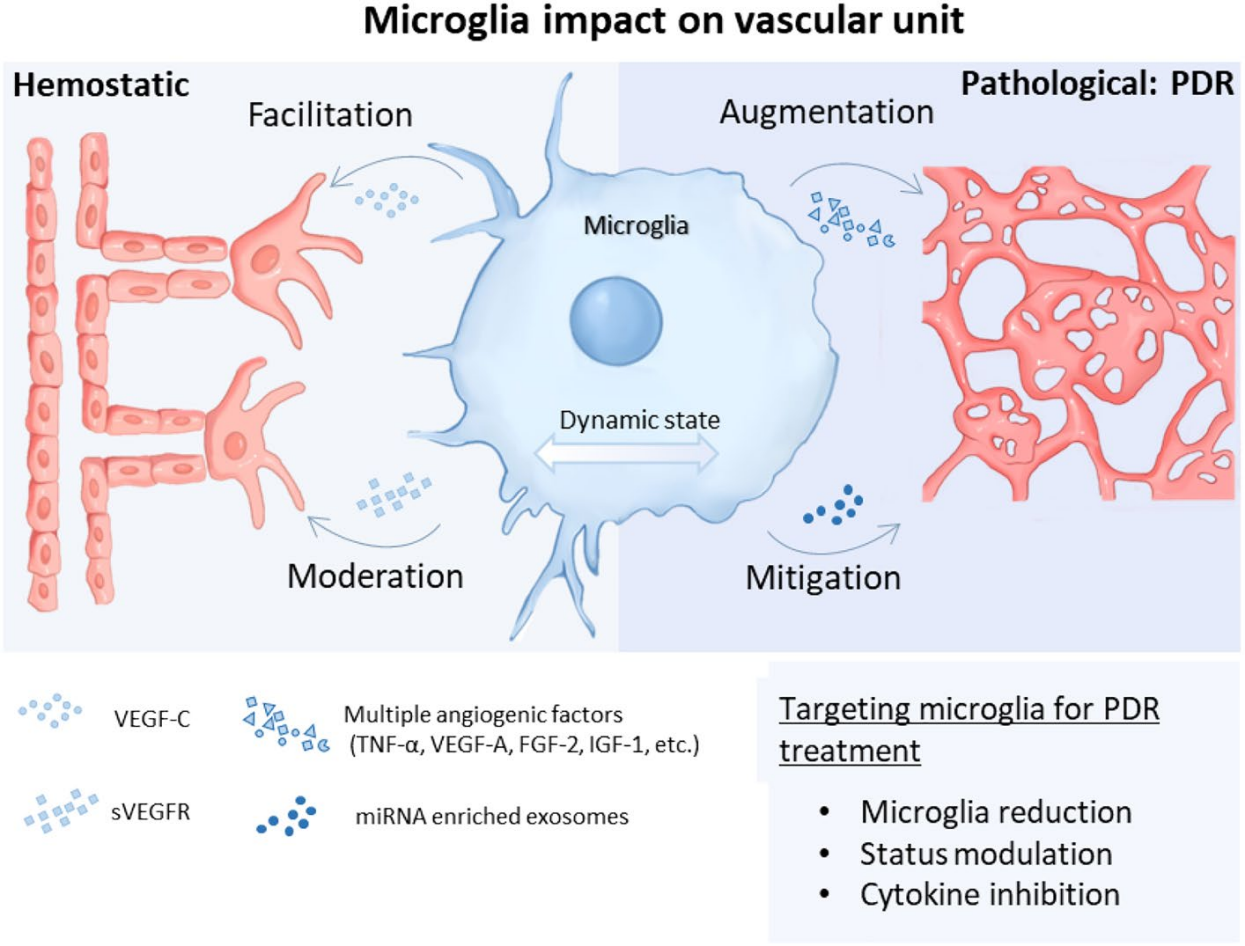

## Introduction

Diabetic retinopathy (DR) is a microvascular complication of diabetes mellitus, which can be classified into non-proliferative and proliferative DR and is receiving more attention due to the increasing prevalence of diabetes. Non-proliferative DR (NPDR) is characterized by microaneurysms, intraretinal hemorrhages or blood vessel blockage, and represents the initial stage of DR. Proliferative DR (PDR) is the advanced stage of DR, during which growth factors stimulate the formation of new blood vessels, a process known as retinal angiogenesis [1]. The development of new blood vessels can cause complications such as visual loss, retinal detachment, and neovascular glaucoma [2]. DR can be graded as non-sight-threatening DR (NSTDR) and sight-threatening DR (STDR). The former includes mild and moderate NPDR, and the latter refers to diabetic maculopathy, severe NPDR, and PDR. Sight-threatening DR (STDR) is the cause of severe visual impairment among working-age adults (20–65 years old) [3]. Around one-third of diabetic patients suffer from DR, with more than two third of those displaying NPDR [4, 5]. Additionally, between 1–2% of type 2 diabetic patients develop severe NPDR/PDR [6]. By 2045, the number of adults worldwide estimated to have DR and vision-threatening DR is projected to reach 103.12 million and 28.54 million, respectively [7]. Although the prevalence of severe NPDR/PDR is lower than that of mild/moderate NPDR, the serious threat to visual ability makes severe NPDR/PDR an important focus of basic and clinical research.

In PDR, retinal neovascularization is driven by ischemia and tissue hypoxia and involves changes in the quantity and status of various retinal cells, which influence retinal neovascularization through secreted factors or direct contact with retinal endothelial cells (ECs) [8]. Neovascularization arises from the retina and either grows along the posterior vitreous, typically found on the optic disc and surrounding area (neovascularization of the disc [NVD]) or along the remaining area of the retina (neovascularization elsewhere [NVE]). Retrospective studies have shown that NVE is the more common pattern of retinal neovascularization in PDR patients, with most NVE located at the posterior pole [9, 10].

PDR has been originally classified as a microvascular disease, characterized by pathological neovascularization. A recent review highlighted the wide involvement of all retinal cells, including ECs and glial cells, in the pathological process of DR [11]. Microglia have been found to play a prominent role in regulating the process of DR through their role in inflammation [11, 12]. By uncovering the involvement of microglia in retinal vasculature development, their role in promoting pathogenic angiogenesis such as the brain and ocular diseases has gained increasing attention [13–16]. In the condition of in vitro hypoxic culture, microglia were induced to express increased angiogenic factors, such as vascular endothelial growth factor (VEGF) and insulin growth factor (IGF-1) [17, 18]. Thus, microglia-mediated neovascularization is of particular interest in the PDR pathophysiology of hypoxic-induced neovascularization.

While current standard-of-care treatments for PDR, such as pan-retinal photocoagulation (PRP), intravitreal injections of anti-vascular endothelial growth factor (anti-VEGF), angiopoietin inhibitors, and vitrectomy are beneficial, they exhibit significant limitations from which patients suffer. These limitations include inadequate or non-responsiveness in a subset of patients, side effects like permanent loss of peripheral vision and exacerbation of macular edema [19–21]. This highlights a high demand for innovative therapeutic strategies. Recently, novel therapeutic modalities for PDR have emerged, such as oxidative stress and inflammation inhibitors or mesenchymal stem cell therapy, pointing towards alternative anti-angiogenesis therapies beyond the scope of current VEGF based approaches [22, 23]. Additionally, microglia-targeted pharmacotherapy has been revealed as a therapeutic strategy in degenerative and inflammatory diseases of the retina such as retinitis pigmentosa and PDR [24, 25]. Studies pointed out targeting microglial cells and their polarization states could be a promising adjunctive therapy for DR, offering a new and alternative avenue for treatment beyond conventional methods [24, 26, 27]. These novel approaches, particularly the modulation of microglia polarization in early DR stages, represent a significant shift from traditional VEGF based treatments, providing new possibilities for managing PDR. This review provided valuable insights into the characteristics of microglia in DR, elucidating their significant influence on pathological angiogenesis in DR. The main mechanisms of microglia in PDR are based on their direct and indirect influences on blood vessel formation. It is therefore necessary to first consider the role of microglia in the physiological development of blood vessels before continuing with pathological condition. The review examined the migration, distribution, and function of microglia during retinal vasculature development, emphasizing their crucial role in regulating physiological blood vessel formation in the retina. Moreover, the review explored the interaction between current angiogenesis inhibition drugs and microglia, highlighting how these drugs, both in clinical practice and laboratory research, modulate microglial activity to inhibit retinal neovascularization in DR. Thus, it not only consolidates existing knowledge but also paves the way for future investigations, highlighting the promising role of microglia as a key therapeutic target in the continuous fight against DR. As we continue to unravel the multifaceted roles of microglia in retinal health and disease, their significance in developing innovative treatment strategies for DR becomes ever more apparent.

## Retinal blood vessel development

The formation of a well-functioning network of blood vessels is essential for the survival and proper functioning of organs. This process involves two steps: vasculogenesis and angiogenesis. Vasculogenesis entails the formation of initial vascular networks by differentiating, expanding, and coalescing endothelial precursors or angioblasts into ECs. Once these networks are established, new capillaries grow via sprouting angiogenesis or intussusceptive angiogenesis [28]. Sprouting angiogenesis involves degrading the basement membrane and activating quiescent ECs into the tip cell, which forms filopodia. The stalk cell then proliferates, and sprouted vessels elongate and anastomose to form the lumen of the sprout. Vessel pruning eventually optimizes the newly formed vasculature. On the other hand, intussusceptive angiogenesis occurs when invagination of tissue into the vascular lumen by transluminal pillars forms new vessels.

**Fig. 1:**
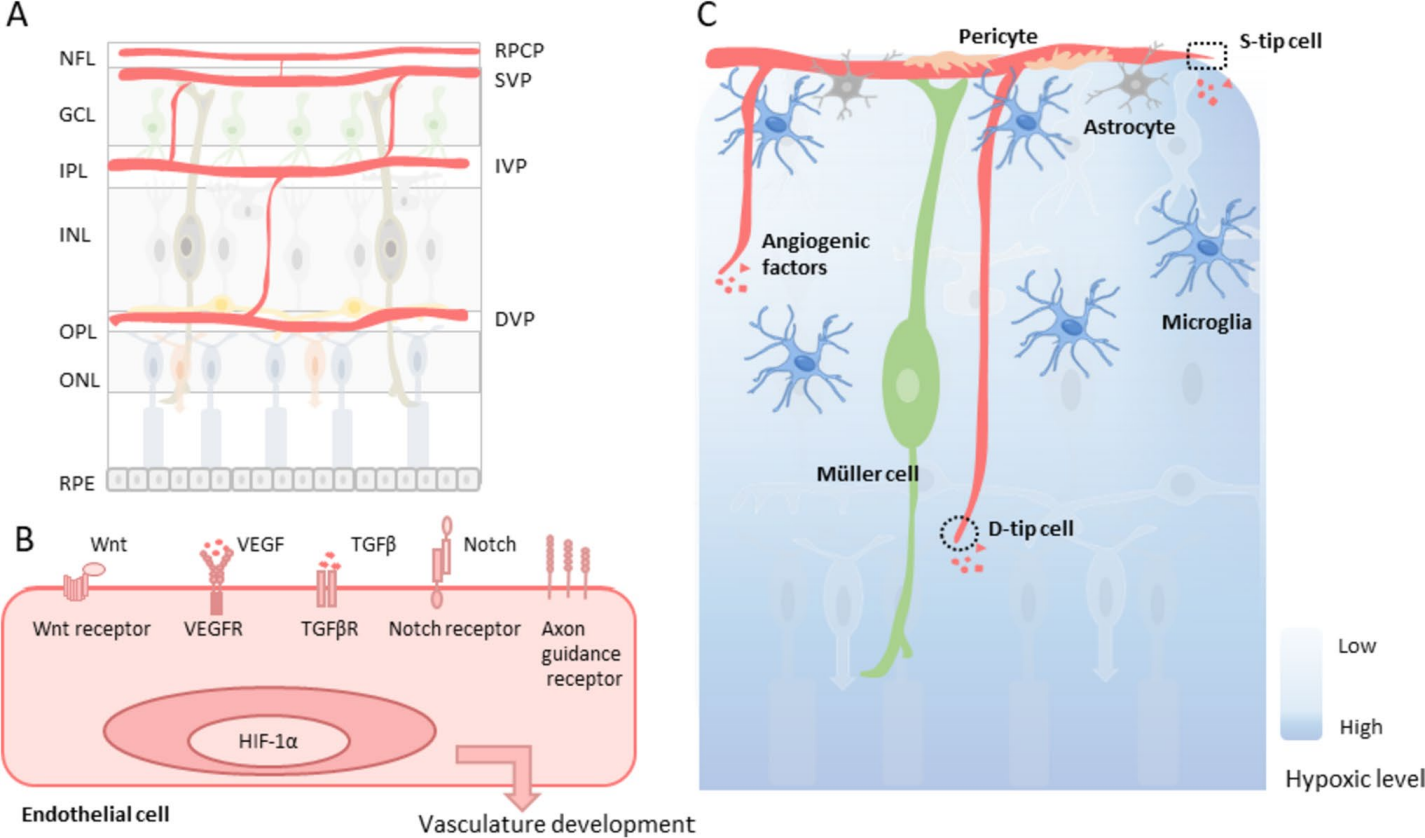
The vasculature of the retina is regulated by a complex interplay of signaling pathways and the cell–cell interaction of different cell types**. A** Mature retinal vasculature. **B** Major cell signaling contributing to retinal vasculature development. **C** Overview of cell types supporting retinal neovessel formation. *RPCP,* radial peripapillary capillary plexus; *SVP,* superficial vascular plexus; *IVP,* intermediate vascular plexus; *DVP,* deep vascular plexus; *NFL,* nerve fiber layer; *GCL,* ganglion cell layers; *IPL,* inner plexiform layer; *INL,* inner nuclear layer; *OPL,* outer plexiform layer; *ONL,* outer nuclear layer; *RPE,* retinal pigment epithelium; *VEGF,* vascular endothelial growth factor; *VEGFR,* VEGF receptor; *TGFβ,* transforming growth factor β; *TGFβR,* TGFβ receptor; HIF-1α, hypoxia-inducible factor 1α

Retinal vasculature development is a highly orchestrated process extensively studied as a proxy for angiogenic processes in other organs, as it allows for easy and simultaneous visualization of different angiogenesis steps. The mature human retinal vasculature is a highly complex network comprising multiple interconnected capillary layers (Fig. 1A). The superficial vascular plexus (SVP) is nestled within the ganglion cell layers (GCL) of the retina. Situated beneath the SVP are two deeper plexuses, the intermediate vascular plexus (IVP) residing close to the top of the inner nuclear layer (INL), and the deep vascular plexus (DVP) lying closer to the bottom. Beyond these three layers, a fourth plexus the radial peripapillary capillary plexus (RPCP) is present, running parallel to the bundles of ganglion cell axons in the nerve fiber layer (NFL) where it helps to meet their high metabolic demands. These capillaries are particularly prominent in the macular area, where axons are most abundant and the nerve fiber layer is thickest [29, 30]. Retinal vasculature development in humans starts around 15 weeks of gestation, with the formation of superficial retinal vessels adjacent to the optic nerve head that extend peripherally. The development of the whole retinal vasculature is completed before birth [31, 32]. In mice, retinal blood vessels develop similarly to humans, but the development starts after birth, involving three layers that lack the radial peripapillary capillary plexus. The superficial vascular network takes approximately one week to reach the periphery, and the superficial capillaries sprout vertically first to form the deep and later the intermediate vascular plexus. Blood vessels reach the periphery of the retina in the deep plexus at postnatal day 12 (P12) and in the intermediate plexus at P15. Three weeks after birth, mouse retinal blood vessels complete all three layers [33–35]. During retinal vasculature development, new vessels are formed mostly by angiogenesis, although vasculogenesis still occurs at a low frequency [36]. Sprouting angiogenesis is responsible for the initial network development, while intussusception contributes to vascular network remodeling and maturation, with these two kinds of angiogenesis often occurring in parallel [37].

Retinal vascular development is regulated by a complex interplay of signaling pathways and different cell types that act in a coordinated manner to ensure the proper formation and remodeling of the retinal vasculature (Fig. 1B). The balanced expression of angiogenic and anti-angiogenic factors promotes new blood vessel development and inhibits excessive neovascularization following the establishment of retinal vasculature. The hypoxic environment stimulates sprouting angiogenesis in the avascular retinal region, via the activation of the hypoxia-inducible factor 1α (HIF-1α) signaling pathway and other angiogenesis-related factors. Hypoxia is more prominent in the peripheral region and deeper layers of the retina, directing blood vessel growth throughout the entire organ. Alongside HIF-1α, other signaling pathways such as the axon guidance pathway [38], Notch and VEGF signaling pathway [39], transforming growth factor-β (TGF-β) signaling pathway [40] and Wnt signaling pathway [41], also contribute to retinal vascular development.

Various cell types and factors play critical roles in regulating retinal vascular development, including tip cells, macroglial cells, and microglia (Fig. 1C). Two types of tip cells, superficial tip cells (S-tip cells) and diving tip cells (D-tip cells) are essential for proper postnatal sprouting angiogenesis. S-tip cells regulate the formation of the superficial layer of vessels, while D-tip cells promote deep vascular plexus formation [42]. As demonstrated recently, pericytes provide structural support to existing vessels by adhering to the abluminal surface of ECs while also contributing to the promotion of EC sprouting [43]. Further, macroglial cells in the retina, such as astrocytes and Müller cells, play a crucial role in supporting vascular development. Astrocytes form a scaffold for assisting in the formation of new blood vessels [44], and loosely sheath the newly formed vessels, working together with Müller cells to maintain the blood-retinal barrier [45]. Lastly, microglia, another kind of neuroglial cell in the retina, support vessel growth and stabilization [46].

## Retinal microglia

Neuroglia were first described in the mid-nineteenth century by Rudolf Virchow, who first identified and characterized non-neuron cells in terms of glia [47]. Initially referred to as "nervous system-specific cells," growing research in the following decades revealed that these cells are specialized immune cells distinct from other brain cells such as neurons and glia. The term "microglia" was first coined by a Spanish neurologist, Pio del Rio-Hortega, who carried out systematic studies of glial cells in the early twentieth century. He described the distribution and morphological phenotype of microglia and considered them as a third element distinct from glial cells and neurons in the brain [48]. Later, the existence and precise morphology of microglia were also described in the retina, distinguishing them from other types of glia and horizontal cells [49–51]. Since then, microglia have been extensively studied in the retina and the brain, and their diverse functions and roles in various disease conditions have been increasingly appreciated.

### Classification of microglia: beyond the M1/M2 paradigm

In the central nervous system (CNS), microglia are unique as specialized immune cells. They are the primary immune effector cells in the CNS, constantly monitoring the microenvironment for abnormal or damaged cells, pathogens, and other foreign substances. In addition to their immune functions, microglia play critical roles in maintaining CNS homeostasis and normal functioning. They are involved in various processes, including synaptic pruning, neural development and repair, neuronal plasticity, and neuroinflammation [52].

Microglia can be classified into distinct subpopulations based on their morphology, gene expression profile, location, and functional state. Historically, microglia have been classified into two main states: the "classically activated" (M1) and "alternatively activated" (M2) phenotypes. This binary classification, borrowed from macrophage biology, was based on the response of these cells to different stimuli: M1 microglia were considered pro-inflammatory, while M2 microglia were deemed anti-inflammatory or reparative. M1 microglia were characterized by the expression of markers such as iNOS, CD16, and CD32, and the production of pro-inflammatory cytokines like interleukines (IL) IL-1β, IL-6, and tumor necrosis factor-α (TNF-α). In contrast, M2 microglia were identified by markers such as CD206, Arginase 1, and Ym1, and were associated with the release of anti-inflammatory cytokines like IL-4 and IL-10 [53, 54]. This framework, however, has been criticized for its oversimplification of the diverse and dynamic nature of microglial responses, especially in the context of complex CNS pathologies. Recent advancements in single-cell RNA sequencing (scRNA-seq) and other high-throughput technologies have revolutionized our understanding of microglial heterogeneity. These innovative tools have enabled a more multifaceted characterization of microglia across various dimensions, including transcriptional, epigenetic, translational, and metabolic profiles [55]. This spectrum ranges from homeostatic states to various activation states induced by pathological conditions such as neurodegeneration, injury, and infection.

In healthy status, microglia maintain a homeostatic state, characterized by a unique transcriptional profile that includes genes like purinergic receptor P2RY12 and C-X3-C motif chemokine receptor 1 (CX3CR1). These cells perform routine surveillance of the brain environment, phagocytosing apoptotic cells and synaptic debris, and supporting neuronal health and synaptic plasticity [56]. In response to neurodegenerative diseases, microglia can adopt a disease-associated phenotype, often referred to as disease-associated microglia (DAM) [57]. Those microglia have been involved in the pathogenesis of neurodegenerative diseases, such as Alzheimer’s disease and retinitis pigmentosa [57, 58]. They have a high diversity and a disease stage-specific transcriptome profile indicating a dynamic response to the disease. The transcriptome is characterized by the upregulation of immune genes such as triggering receptor expressed on myeloid cells 2 (Trem2), apolipoprotein e (Apoe) and cystatin 7 (Cst-7) and downregulation of homeostatic genes such as P2ry12 and transmembrane protein 119 (Tmem119) [59]. Following CNS injury or infection, microglia rapidly become reactive, undergoing morphological changes and upregulating genes involved in inflammation and immune responses. This state is more similar to the traditional M1 phenotype but is more complex, involving a range of pro-inflammatory and anti-inflammatory mediators, as well as neurotrophic factors [53, 54, 60].

While both brain and retinal microglia are developed from the primitive yolk sac, the characteristics vary depending on the region of the CNS. In spite of the differences, retinal and brain microglia have been found to share the expression of several transcription factors. Retinal microglia exhibit unique characteristics that are pivotal for retinal health and pathology. Although, most of the knowledge about microglia came from brain studies, scRNA-seq of retina allowed a more in-depth understanding of the heterogeneity of the retinal microglia populations. These analyzes identified four distinct clusters of retinal microglia (RMG), designated as RMG0, RMG1, RMG2, and RMG3. RMG0 and RMG2 are clusters primarily associated with microglial homeostasis in the retina, expressing genes indicative of surveillance and support functions. In contrast, RMG1 microglia are significantly enriched in disease conditions, such as oxygen-induced retinopathy (OIR) mouse model, suggesting an enhanced pro-inflammatory response during retinal ischemia. Concurrently, RMG3 microglia exhibit upregulation of angiogenesis-related genes, implicating their role in neovascularization processes, both pathological and reparative [61]. Thus, these findings revealed microglia’s heterogeneity and underscored the complexity of microglial functions in the retina, going beyond simplistic classifications and highlighting their adaptive roles under various retinal conditions.

### Identification of microglia in retina

Retinal microglia display various phenotypic characteristics and molecular markers throughout their development, thus identifying these cells relies on a broad range of markers using transcriptomics and proteomics. Brain and retinal microglia exhibit high similarity due to their shared extra-embryonic yolk sac origin, low turnover rate, and minimal contribution from blood monocytes. Additionally, they share a large transcriptional overlay, and a comparable set of factors specific to microglia lineage are also present in the retinal microglia [62]. Markers of CNS microglia in the brain have been extensively studied and exhibit specific marker expression profiles in different developmental stages [63], but there is a deficiency in comparing microglia markers horizontally over time in the retina.

The identification of retinal microglia has been refined over the years, moving from general myeloid markers to more specific microglial signatures. Initially, microglia were identified in the retina using common myeloid markers such as CD11b, Iba1, and CD68, shared with macrophages and other myeloid cells [64]. CD11b and ionized calcium-binding adapter molecule 1 (Iba1) are pan-microglial markers extensively used to identify microglia in various states, while CD68 is associated with phagocytic activity. However, these markers are not exclusive to microglia, necessitating the use of more specific markers for precise identification.

Extensive research has led to the identification of microglia-specific markers, such as TMEM119 and P2RY12 [65]. TMEM119 is exclusively expressed by microglia in the CNS and is absent in other myeloid cells, making it a reliable marker for microglial identification. P2RY12, a purinergic receptor, is involved in microglial surveillance functions and is also considered a specific marker for resting microglia. To the current knowledge, the most specific microglia markers are TMEM119 and P2RY12. Additionally, hexosaminidase subunit beta (HEXB) has been identified as a specific marker for microglia, further aiding in their precise identification and differentiation from other myeloid cells [62, 66].

Besides transcriptomics and proteomics, the morphological characteristics of retinal microglia also provide insights into their identification and functional states. Morphologically, healthy retinal microglia display a ramified form, changing to an amoeboid shape during activation in response to retinal injury or disease. Advanced imaging techniques, such as two-photon microscopy, have been instrumental in visualizing these morphological changes in vivo, providing a dynamic view of microglial behavior in the retinal environment [67].

In conclusion, the identification of retinal microglia has evolved significantly, with the adoption of specific molecular markers and advanced imaging techniques providing a more accurate and nuanced understanding of these cells. The use of these specific markers helps in the precise identification of microglia and understanding of their functions across various developmental stages in the retina.

### Occupation and distribution of microglia in healthy retina

The occupation of microglia in the retina occurs before the development of blood vessels. Studies of the human fetal retina indicated that microglia already occupied the entire surface of the retina at 15 weeks gestation, while the retinal blood vessels only originated from the optic disc [46]. In mice, microglia appear in the peripheral regions of the retina at an early embryonic age of 12.5 days [68]. Within the first postnatal week, microglia are present as singular cells ahead of the growing vascular plexus [69]. The migration of microglia into and within the retina is regulated by various cytokines and other factors. During the migration of embryonic microglial precursors into the developing retina, blood vessels act as scaffolds. The cytokine signal, initiated by IL-34, triggers microglia precursor movement from the yolk sack towards the retina. Hyaloid blood vessels act as scaffolds for migration as microglia enter the optic cup. The neurogenic state of the retinal tissue subsequently serves as an entry signal during the infiltration of microglia into the retina [70]. Further proteins that are essential for the physiological migration and recruitment of microglia in the neonatal retina are stromal cell-derived factor-1 (SDF-1) and its receptor CXCR4. In situ hybridization studies revealed that SDF-1 has been expressed in retinal arterioles, while CXCR4 exhibited spot-like signals in specific subpopulations of retinal microglia. The inhibition of SDF-1 or CXCR4 reduced the movement of microglia [71].

**Fig. 2:**
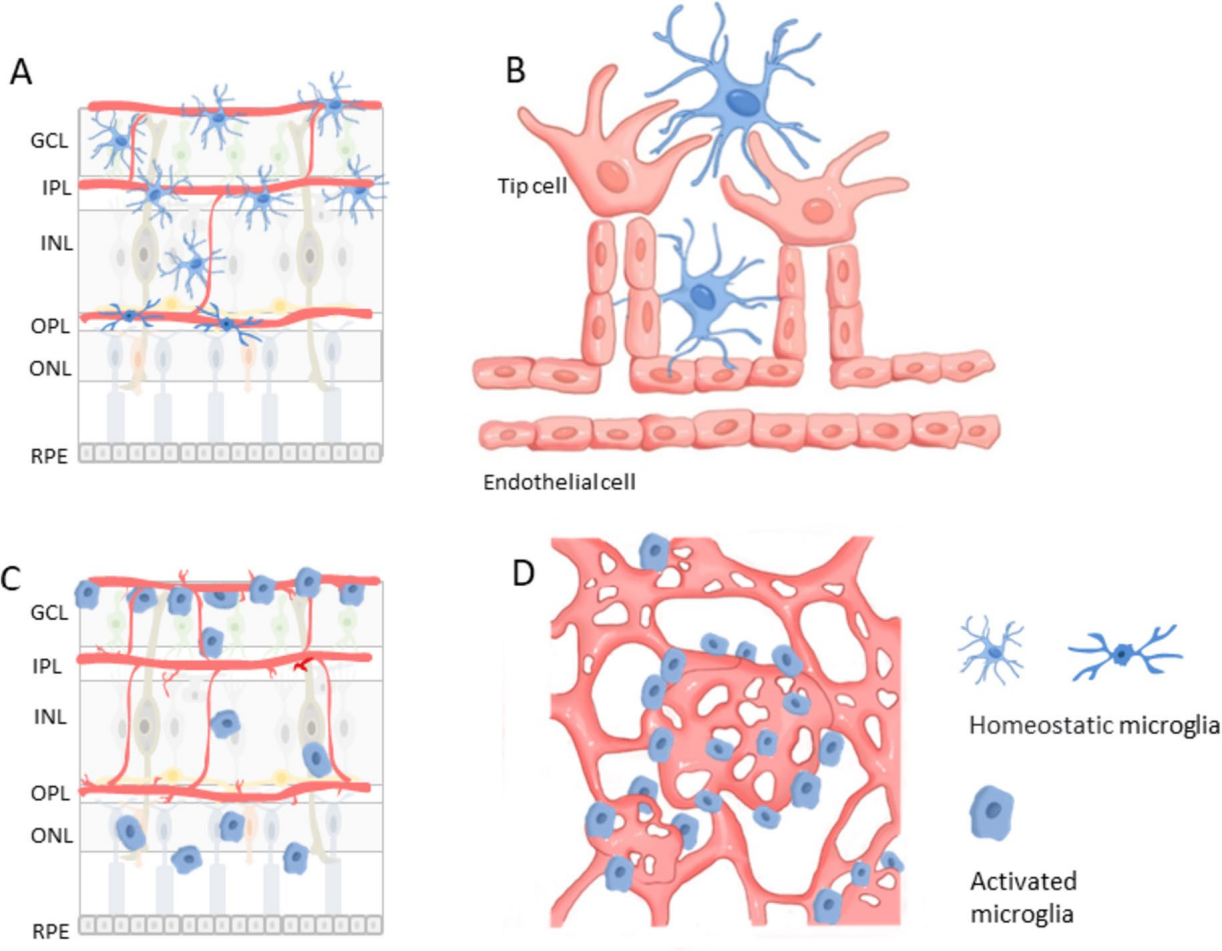
Distribution of microglia in the retina. **A** Microglia distribution and morphology in different layers of the retina in physiological conditions. **B** Microglia distribution and morphology at sprouting site in physiological condition. **C** Microglia distribution and morphology in different layers of the retina in diabetic retinopathy.** D** Microglia distribution at neovasculature tufts in diabetic retinopathy. *GCL,* ganglion cell layers; *IPL,* inner plexiform layer; *INL,* inner nuclear layer; *OPL,* outer plexiform layer; *ONL,* outer nuclear layer; *RPE,* retinal pigment epithelium

During the first week, microglia mainly appear in the superficial vascular layer, and from P8 to P13, they progressively migrate through different retinal layers from the ganglion cell layer to the outer plexiform layer, directed by the increasing retina stiffness [72]. During this process, the shape of microglia changes in each layer. They display a ramified shape in the softer outer plexiform layer, indicating their surveillance and sampling functions. In contrast, microglia become bipolar rod-shaped within the stiffer outer nuclear layer, where they tightly wrap around the blood vessels, indicating their role in regulating retinal vascular development. However, the mechanism of stiffness recruitment is not yet clear, but it appears to be integrin-independent [73]. After the completion of retina vascularization, microglia settle within almost the entire retina (Fig. 2A) including the retinal ganglion cell layer, inner plexiform layer, inner nuclear layer, and outer plexiform layer [36, 64, 68, 74]. The density and distribution of microglia in the murine retina are closely linked to vascular growth. After birth, the density of microglia decreases in the entire retina. However, during the first week after birth, the density increases because microglia precursors enter from the vitreous. Subsequently, the density decreases again as microglia entry ceases and the retina expands in size [64, 68]. During the first week after birth, retinal vascularization begins to occur, and endothelial tip cells start to extend outwards to form vascular sprouts. At this stage, microglia are evenly distributed at a low density since the retina is mainly avascular. As vascularization progresses, microglia slightly accumulate around the newly vascularized center. In P5, the microglia show higher density and more amoeboid morphology in the vascular area compared to the avascular (peripheral) area, where microglia appear to be more dispersed [75]. Microglia are densely distributed around blood vessels in the inner retina, forming a surrounding network-like structure and interacting with endothelial tip cells along the leading edge of the sprouts (Fig. 2B), guiding and regulating the anastomose [69]. During this stage (P0–P7), microglia density in the inner vascular retina increases, while it remains relatively constant in the outer avascular retina during this time [76]. By the end of the first week, as the superficial layer of the whole retina is vascularized, microglia density becomes equal in the central and peripheral regions [77]. During the mid-postnatal stage (P7–P21), retinal vascularization continues, leading to the completion of the deep capillary network and dual capillary networks. At this stage, microglia are more closely associated with endothelial sprouts and their morphology changes to a polarized form that extends along the leading edge of the growing vessels. In the late postnatal stage (over 21 days) and adult stage (P60), retinal vasculature reaches maturity, and microglia are distributed throughout the retina, with a lower density around blood vessels [64, 78]. Finally, in the mature retina, microglia comprise only 0.7% of all retinal cells and less than 10% of all glia but more than 80% of retinal immune cells [79, 80].

## Microglia in retinal blood vessel development: neovascular facilitation and moderation

Microglia play a critical role in maintaining proper retinal vascular development. They help to connect neighboring sprouts and promote vessel fusion, leading to the formation of a robust vascular network [76, 81]. A genetic deficiency of microglia can reduce the contact points between neighboring tip cells during early retinal development, but it is compensated by a decrease in vessel pruning in later stages [69, 82]. Moreover, microglia guide the developing blood vessels’ direction, and their displacement causes the vessels to grow in the wrong direction [74]. The influences of microglia towards retinal vasculature at different stages are oppositional: angiogenic in the early stage but anti-angiogenic in the late stage when vascular layers are entirely formed. Pharmacological depletion of microglia during the embryonic stage [83] or first postnatal week [84] can cause severe capillary dropout and disappearance in the established vascular area. Interestingly, the administration of microglia depletion drugs starting from P9 led to a slightly increased vascular density in P18, indicating that microglia’s influence on retinal deep layer vasculature is inhibitory. Further research has identified a specific subpopulation of microglia responsible for inhibiting excessive branching [76, 85].

### Microglia as promoters of vascular growth

The function of microglia in regulating blood vessel development is mainly based on two factors: first, through the direct interaction with ECs, and second, through indirect interactions such as the secretion of soluble effectors (cytokines and growth factors).

**Fig. 3:**
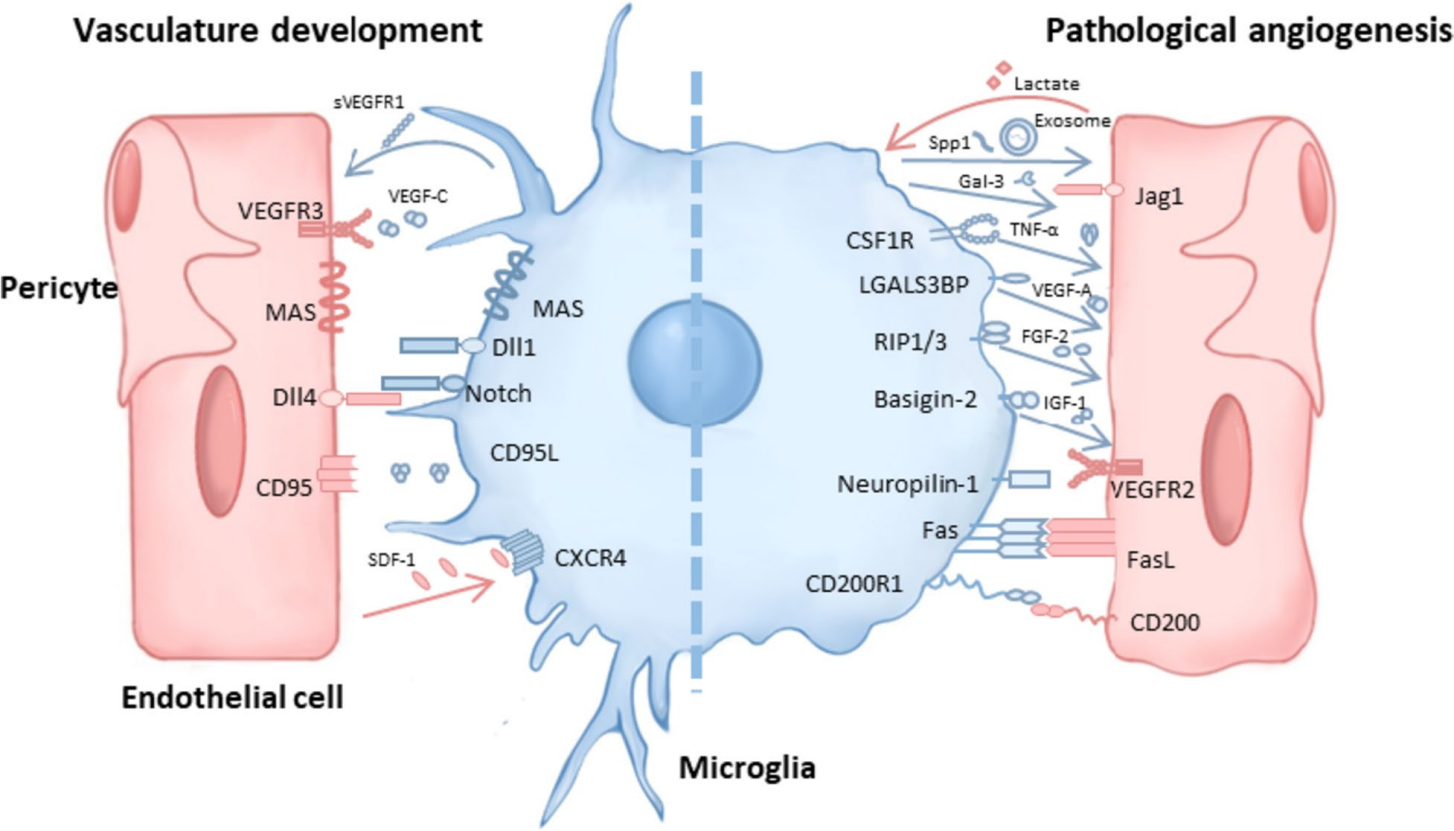
Molecular interactions between microglia and ECs. Microglia govern retinal vasculature development via interaction with ECs through receptor-ligand pairs or the secretion of soluble factors (shown in the panel to the left). In DR, microglia alter their ligand expression pattern or secrete angiogenic factors in order to influence pathogenic angiogenesis. Additionally, ECs alter the microglia phenotype as well as microglia polarization (shown in the panel to the right). sVEGFR1, soluble vascular endothelial growth factor receptor 1; VEGF-C, vascular endothelial growth factor C; VEGFR3, vascular endothelial growth factor receptor 3; Dll1, delta-like ligand 1; Dll4, delta-like ligand 4; CD95L, CD95 ligand; CXCR4, C-X-C chemokine receptor type 4; SDF-1, stromal cell derived factor 1; Spp1, secreted phosphoprotein 1; Gal3, galectin 3; Jag1, jagged1; CSF1R, colony stimulating factor 1 receptor; TNF-α, tumor necrosis factor alpha; LGALS3BP, lectin galactoside binding soluble 3 binding protein; RIP1/3, receptor-interacting serine/threonine-protein kinase 1/3; FGF2, fibroblast growth factor 2; IGF-1, insulin-like growth factor 1; NRP-1, neuropilin-1; FasL, Fas ligand; CD200R1, CD200 receptor 1

Both mechanisms are guided by many different molecules, which are part of several signaling pathways. One essential pathway is the Notch pathway (Fig. 3). Tip cells positive for the Notch ligand delta-like 4 (Dll4) activate Notch signaling in microglia situated between them. Subsequently, Notch-positive microglia act as a bridge between two Dll4-positive tip cells, guiding and promoting ECs to anastomose [81]. Many more membrane surface proteins on microglia are crucial for regulating retinal angiogenesis since their inhibition can impair blood vessel development. MAS, a G protein-coupled receptor binding to the angiotensin II metabolite angiotensin, is essential for microglia recruitment and blood vessel growth at the vascular front of the developing retina. In comparison to wild-type littermate retinas, neonatal mouse retinas lacking MAS exhibited a decreased amount of filopodia and shorter vascular migratory lengths, along with fewer microglia observed at the vascular front [86]. Further, CXCR4-positive microglia have been reported to be recruited by SDF-1, playing a role in promoting neovascular sprouting [71].

Besides the direct interaction between microglia and ECs, microglia-derived soluble factors have been found to enhance sprouting and branching *in vitro*. However, the exact angiogenic factors responsible for this effect remain unclear, although they are known to be distinct from VEGF-A [69]. Consistent with these findings, *in vivo* experiments have demonstrated that during retinal vascular development, VEGF expression was primarily localized in astrocytes rather than in microglia [87]. CD95 and CD95 ligand (CD95L), also referred to as Fas and Fas ligand (FasL), were originally identified in apoptotic cell lymphocytes. In the retina, microglia are the central source of soluble CD95L, which in turn may stimulate CD95 on vessels and support vascular growth mediated by Src-family kinase and PI3K signaling. Microglia-specific deletion of CD95L reduced the neurovascular complexity [88].

Furthermore, microglia also indirectly regulate the development of blood vessels by facilitating the spatial pattern of astrocytes [89]. Astrocytes serve as a scaffold during the retinal vascular development. Depletion of microglia in mice has been associated with defective astrocyte templates, along with a significant reduction in the retinal vascularized area [83].

### Microglia as regulators of vascular stability

Microglia not only promote the development of new blood vessels but also have an anti-angiogenic role in maintaining blood vessel homeostasis. VEGF-C positive microglia, located in vessel anastomoses, activate VEGFR-3 in tip cells. This activation led to the expression of Notch target genes and reduced the cells’ sensitivity to the VEGF gradient. As a result, excessive angiogenesis has been prevented [90]. Microglia expressing Notch ligand delta-like 1 (Dll1) restricted vascular branching (Fig. 3), while the conditional deletion of Dll1 in retinal microglia resulted in increased branching [76]. Additionally, microglia can inhibit vascular development by expressing the soluble VEGF inhibitory receptor (sVEGFR), which impedes vascular branching in the deep-layer vasculature [85].

In summary, microglia play a crucial role in the development of retinal vasculature. They are not only located in close proximity to blood vessels but also interact with ECs through the release of soluble effectors. Microglia enhance angiogenesis during the early stages and later inhibit angiogenesis while promoting vessel pruning, emphasizing their importance in establishing a proper vasculature network within the retina.

## Microglia in diabetic retinopathy (DR): angiogenic augmentation and mitigation

PDR is the advanced stage of DR, characterized by the proliferation of new blood vessels in the retina, which can lead to hemorrhage, macular edema, and if left untreated, blindness [91]. The pathogenesis of PDR is closely linked to the buildup of advanced glycation end products (AGEs), which activate the receptor for AGEs (RAGE), contributing to the development of PDR [92]. This activation triggers an increase in EC proliferation, angiogenesis, and the expression of pro-angiogenic genes, including Angiopoietin 1/2 (Ang1/2), VEGF, VEGFR, platelet-derived growth factor (PDGF), and fibroblast growth factor (FGF) [93]. Furthermore, AGE-promoted angiogenesis is accompanied by reduced pericyte coverage and disorganized endothelial alignment in microvessels [94]. The angiogenesis is further exaggerated by the activation of microglia, which produce pro-inflammative and angiogenic cytokines, including IL-1β, TNF-α, and VEGF-A. Notably, there is emerging evidence suggesting that microglia might express VEGFR1 which is activated under pathological conditions and further might upregulate proinflammatory and pro-angiogenic cytokines such as VEGF-A and TNF-α, placental growth factor (PGF) [87]. However, the effects described to date in the literature are based on a mixed population of microglia and macrophages. Nevertheless, the microglia VEGFR1 pathway and the downstream effects may have a dramatic influence on pathologic blood vessel development in the retina. Hypoxia, which is commonly observed in PDR, further exacerbates the inflammation and angiogenic response, contributing to retinal neovascularization. This link between inflammation, hypoxia, and angiogenesis ultimately results in the formation of new, abnormal blood vessels in the retina, which may cause retinal detachment, macular edema, and vision loss in PDR patients [95].

Animal models have played a crucial role in advancing our understanding of the pathogenesis and treatment of neovascularization in DR. Among the various models, the oxygen-induced retinopathy (OIR) model is most frequently employed. This model involves exposing neonatal rats and mice to hyperoxia and then returning them to normoxia, leading to the development of zones of retinal ischemia and hypoxia. Consequently, neovascularization is induced, simulating the ischemic component of DR [96].

### Alteration of microglia quantity, distribution, and morphology in DR

One of the cell populations that undergoes a significant alteration in hypoxia-induced retinopathy are microglia, which exhibit changes in their quantity, distribution, and morphology within the retina in DR, compared to healthy controls. In the OIR mouse model, microglia density increased around P12, when neovascularization development begins. At P17, when neovascularization peaks, the maximum density of microglia has been observed in the superficial vascular layer of the retina [97]. Further, during neovascularization the microglia density has been upregulated in OIR model compared to healthy control, which is likely due to increased neovascularization [97]. Microglia in the neovascularization area showed mostly an activated status with hypertrophic amoeboid morphology. More than 80% of microglia in the avascular and neovascularization tuft region have been detected to be activated, while less than 2% have been activated under normal conditions [98]. In the OIR mouse model, studies based on the obsolete M1/M2 classification have shown that, both M1 and M2 microglia populations exist. Their percentages undergo dynamic changes. During the early stage of neovascularization, microglia that accumulate in neovascularization tufts are majorly M1 polarized, whereas during the late stage (after P20), M2 polarization of microglia gradually increases, inhibiting the neovascularization and promoting the recession of neovascularization tufts [99]. In compliance with mouse models, the alteration in microglia quantity and activation status arised in DR patients, with a significantly increased number of microglia detected in diabetic patients before they progressed into DR [100]. Furthermore, microglia have been already activated before the advanced stage of PDR, as amoeboid-like microglia have been detected in NPDR patients’ retinas [101].

Unlike the normal condition, in which microglia are found deepest in the outer plexiform layer, microglia are detected in the inner nuclear layer and outer nuclear layer in the OIR mouse model [102]. In DR patients’ retinas, microglia accumulate more around dilated veins, microaneurysms, intraretinal hemorrhages, cotton-wool spots, and retinal neovascularization, even migrating to the outer retina and epiretinal membrane (Fig. 2C) [103]. Focusing on the localization of microglia in DR (Fig. 2D), the activated microglia have been observed in the central avascular area, mid-peripheral neovascular tufts, and peripheral normal vascular area and especially accumulate in and around pathological retinal angiogenic niches [101, 104, 105].

The increase in microglia observed in the neovascularization area was initially attributed to the recruitment of these cells from non-neovascularization areas, driven by chemokines like C–C motif chemokine ligand 2 (CCL-2). CCL-2, secreted by ECs and activated microglia, plays a crucial role in this recruitment process [106]. Indeed, the absence of CCL-2 has been shown to result in a reduced number of microglia in neovascularization lesions [107]. However, debate still exists over whether the overall increase in myeloid cells in the OIR model and the accumulation in the neovascularization area is caused by the local proliferation of resident microglia or the migration of blood-derived macrophages. Boeck et al. observed increased microglial proliferation in the inner retina and neovasculature using EdU staining, while Dejda et al., through flow cytometry, found no notable proliferation differences in mononuclear phagocytes between normoxia and OIR [102, 108]. The discussion around the specific roles of resident microglia and invading monocytes in degenerative retinal diseases is extensive [109, 110]. However, it remains inconclusive for DR. Many studies still lack reliable markers to effectively differentiate between microglia and macrophages. Reliable markers solely expressed by microglia are P2RY12 and TMEM119. Consequently, some researchers use broader terms like retinal myeloid cells or a combined microglia/macrophage category, which fails to precisely clarify the individual contributions of these cells in DR, particularly regarding their involvement in pathogenic angiogenesis, thereby highlighting a critical gap in the current research and understanding of the unique roles of invading monocytes in DR. Nevertheless, these studies indicated that the accumulation of microglia in the neovascularization region was partially caused by the increased proliferation of resident microglia. Further mechanistic investigations are required to better understand microglia accumulation in neovascularization lesions and distinguish the contribution by resident microglia and invading monocytes.

### *Activation and angiogenic phenotype of microglia *in vitro

Microglia accumulate in DR to significantly affect pathogenic angiogenesis and contribute to angiogenic augmentation. In response to DR, microglia undergo a transformation into an angiogenic phenotype, which enables them to secrete pro-angiogenic factors. This transformation of activated microglia under disease conditions has been consistently observed in vitro and in DR models in vivo.

In vitro studies revealed that the activation of microglia in a hypoxic and inflammatory environment can lead to their transformation into an angiogenic phenotype. In vitro models using lipopolysaccharide (LPS) and hypoxic culture conditions simulated the inflammatory and hypoxic environment of PDR by activating microglia in a similar manner to what occurs in the disease condition. In hypoxic cultures, retinal microglia expressed an increased level of angiogenic proteins, such as VEGF, interferon-γ,  IL-6, and tissue inhibitor of metalloproteinase 1 (TIMP-1), HIF-1α, VEGF-A, matrix metalloproteinase (MMP)-2, and MMP-9 [17, 18, 111]. They showed that the conditioned media of hypoxic-cultured microglia significantly stimulated the tube formation of ECs, indicating their pro-angiogenic effects. In addition, microglia that have been activated by LPS stimulated the expression of TNF-α and IL-1β, which consequently induced the expression of VEGF-A and PDGF-BB in co-cultured ECs, enhancing angiogenesis, migration, proliferation, and permeability of retinal microvascular ECs that were co-cultured [112]. These microglia were also induced to produce reactive oxygen species, which in turn led to apoptosis in pericytes that were cultured together and may destroy the integrity of the vasculature in vivo [113]. These findings suggest that microglia may play a significant role in promoting angiogenesis in the hypoxic and inflammatory retina by secreting pro-angiogenic factors (Table 1).

**Table 1:** Angiogenic factors secreted by microglia in diabetic retinopathy

Secreted cytokine	DR model	Refs.
Vascular endothelial growth factor A (VEGF-A)	Hypoxia in vitro & in vivo, LPS stimulation	[17, 18, 111, 112]
Tumor necrosis factor α (TNF-α)	LPS stimulation in vitro and hypoxia in vivo	[112, 114]
Interleukin 1 β (IL-1β)	LPS stimulation in vitro	[104, 112]
Interleukin 6 (IL-6)	Hypoxia in vitro	[17]
Tissue inhibitor of metalloproteinase-1 (TIMP-1)	Hypoxia in vitro	[17]
Interferon γ (IFN-γ)	Hypoxia in vitro	[17]
Matrix metalloproteinase 2 (MMP-2)	Hypoxia in vitro	[111]
Matrix metalloproteinase 9 (MMP-9)	Hypoxia in vitro	[104, 111]
Insulin-like growth factor 1(IGF-1)	Hypoxia in vitro and OIR model in vivo	[18, 115]
Fibroblast growth factor 2 (FGF-2)	OIR model in vivo	[116]
Osteopontin	OIR model in vivo	[117]
Galectin 3	OIR model in vivo	[118]

### *Microglia in DR models *in vivo

#### Factor secretion of angiogenic phenotype

Consistent with data obtained in DR models in vitro, microglia in the more physiological OIR models in vivo changed towards an angiogenic phenotype to stimulate neovascularisation. As soon as microglia accumulate around neovascularization tufts, they express more angiogenic factors. These angiogenic phenotypes are associated with a unique expression pattern of surface markers, resulting in distinct microglia subtypes. One subgroup underwent necroptosis by activating receptor interacting protein 1 and 3 signaling (RIP-1 + RIP-3). These necroptotic microglia released fibroblast growth factor 2 (FGF-2), inducing retinal neovascularization [116]. Another subgroup of microglia stained positive for the colony stimulating factor 1 receptor (CSF1R), was morphologically different from resident microglia, and closely associated with retinal blood vessels. This population expressed high levels of TNF-α, which can induce retinal angiogenesis [114]. Basigin 2, an extracellular matrix metalloproteinase inducer, has been enriched in retinal microglia near angiogenic sprouts, and its upregulation in microglia mediated retinal neovascularization by prompting microglia to secrete more insulin like growth factor 1 (IGF-1) [18]. LGALS3BP, another secreted factor and scavenger receptor cysteine rich (SRCR) domain protein, was highly expressed in microglia surrounding regions of neovascularization. Inhibition of LGALS3B impaired the expression of angiogenic factors such as HIF-1α, VEGF-A, or MMP-2 and MMP-9 in microglia-derived cell lines [111]. CD200, a type 1 glycoprotein of the immunoglobulin superfamily, and its receptor CD200R1 were co-localized on vascular ECs in the neovascularization area. The enhanced interaction between CD200R1 on microglia and CD200 on retinal ECs may regulate retinal neovascularization since blockage of CD200R1 inhibits proliferation and activation of microglia and shrinks the pathological neovascularization area [119]. Neuropilin 1 (NRP-1) is a transmembrane glycoprotein that binds angiogenic factors such as VEGF and PDGF. In the central nervous system, NRP-1 is expressed by neurons, ECs, and microglia. NRP-1 in microglia is not necessary for retinal vasculature development but indispensable for pathologic retinal angiogenesis in the OIR retina. The microglia-specific knock out of NRP-1 did not impair the development of mouse retinal vasculature [120]. However, deficiency in NRP-1 led to a decrease in microglia infiltration in the neovascularization lesions and consequently, reduced pathological neovascularization [108].

In addition to surface protein, the microglia’s angiogenic phenotype can be classified according to their changes in transcription factors to stimulate the secretion of angiogenic factors. For example, the non-histone protein Yin Yang 1 (YY-1) was hyperlactylated in microglia in the OIR model, which directly enhanced FGF-2 transcription and, as such, contributed to neovascularization [121].

However, not all studies explicitly distinguish among different microglia status or subgroups. Nevertheless, they secrete various proteins that have the potential for endothelial metabolism in the OIR model. One such protein is secreted phosphoprotein 1 (Spp-1), also known as osteopontin, which has been found to be specifically elevated in microglia retinal neovascularization in mice. Spp1 appears to mediate the communication between microglia and ECs, promoting EC proliferation, which can contribute to pathological neovascularization [117]. Additionally, it has been reported that microglia-derived galectin 3 (Gal-3) binds the Notch ligand jagged1 (Jag-1), blocks the Notch signal and thus promotes EC proliferation (Fig. 3) [118]. Treatment with neutralizing antibodies against both Spp1 and Gal3 has been found to alleviate pathological neovascularization. Thus, multiple subtypes of microglia exacerbate retinal neovascularization by secreting various angiogenic factors and inhibition or depletion of these microglia significantly alleviated neovascularization.

#### Factor secretion depending on polarization

In addition to the factors secreted by microglia subgroups, the progression of DR essentially depends on the polarization status of microglia. Activated microglia can modify their polarization status to secrete angiogenic factors, which are closely linked to angiogenesis in the OIR mouse model. Microglia shift to the M1 polarized state to promote hypoxia-induced angiogenesis [122], while M2 microglia an anti-inflammatory cell type encourage normal angiogenesis and neovascularization regression [123]. M1 microglia have been revealed to induce resting microglia activation via exosome secretion, which ultimately induced abnormal retinal EC proliferation [124]. Retinal ECs secreted glycolytic metabolites, particularly lactate, to induce hyperglycolytic microglia PRAGMs (pathological retinal angiogenesis-associated glycolytic macrophages/microglia). These hyperglycolytic microglia displayed a mixed M1/M2 phenotype and produced pro-angiogenic cytokines such as MMP-9 and IL-1β. They also enhanced retinal EC glycolysis, leading to increased sprouting and proliferation, which ultimately exacerbated the development of pathological neovascularization [104]. A more recent study confirmed the existence of this hyperglycolytic microglia population and highlighted its unique metabolism and close link to retinal pathogenic angiogenesis [125]. As described above, certain microglia with specific polarization statuses have been identified as regulators of the metabolism of retinal ECs.

#### Microglia interplay with neurovascular unit

In the retina, the neurovascular unit is essential for maintaining homeostasis and modulating neuronal activity. Comprising neurons, glial cells (Müller cells, astrocytes, microglia), and vascular elements (ECs, pericytes), its balance is critical. Disruption in the balance and interaction among these components contributes to DR pathogenesis [126]. Neuronal stress, glial activation, and vascular dysfunction lead to blood-retinal barrier breakdown and inflammatory infiltration, creating a neuroinflammatory milieu. Activated microglia exacerbate this by releasing pro-inflammatory cytokines like IL-1β, inducing VEGF production from Müller cells, which indirectly triggers pathological angiogenesis [127]. Concurrently, microglia-induced oxidative stress and reactive oxygen species production further compromise the blood-retinal barrier, creating a cycle that exacerbates the condition [128]. Furthermore, microglia’s interaction with capillaries and pericytes, leading to vascular constriction, could indirectly contribute to retinal neovascularization [129]. Mills et al. found that in the early stage of DR, fractalkine CX3CR1 activation between neurons and microglia led to increased vascular constriction and reduced blood flow [130]. Although this group did not establish a link between microglia-induced reduced blood flow and retinal neovascularization in the DR context, it has been reported that in the retina, decreased blood flow triggered pro-angiogenic signals [131]. Therefore, in addition to their direct impact on endothelial cells, microglia are integral in perpetuating the cycle of neurovascular dysfunction. Their interactions within the neurovascular unit contribute to the progression of pathogenic retinal neovascularization in DR.

#### Anti-angiogenic microglia

As a highly heterogeneous and dynamic population, microglia also play a role as a mediator to mitigate pathogenic angiogenesis instead of promoting it. Studies have shown that depletion of 50% of retinal microglia during both pre-hyperoxia (P8) and post-hyperoxia stages (P12) in the OIR mouse model led to an increase in neovascularization in mid-per areas, particularly during the proliferation stage [96]. Further studies suggested the possible beneficial role of microglia in removing neovascular tufts during the proliferative stage of the OIR. Hematopoietic stem cells (HSCs), which were injected intravitreal, differentiated into microglia-like cells and exerted anti-angiogenic roles. The formation of neovascular tufts was reduced and they promoted the recovery of retinal vasculature in the central avascular area [84]. However, differentiation into microglia-like cells appears to be possible only under pathological conditions with abnormal vasculature where HSCs can engraft the CNS. After differentiation, the transcriptome of these microglia-like cells is similar to that of tissue-resident microglia [132]. Further studies suggested that the therapeutic efficacy of these cells may partially derive from their capacity to protect retinal astrocytes, indicating a potential synergistic interplay between myeloid progenitor cells and astrocytes in normalizing retinal revascularization [133]. One possible mechanism by which microglia may remove neovascular tufts during OIR is by inducing EC apoptosis via two interacting proteins, Fas and FasL. FasL-expressing microglia induced the apoptosis of Fas-expressing ECs and, as resident macrophages of the retina, phagocytosed and removed dead ECs [106]. Alternatively, microglia inhibited EC proliferation and migration. This has been demonstrated by a study showing that thrombospondin-1 (Trp-1) positive microglia retarded EC proliferation and migration through secretion of microRNA enriched exosomes and stimulation of SMAD family member 3 (Smad-3) in ECs [61]. In summary, microglia have a complex role in retinal neovascularization in DR (Fig. 3). Under hypoxic and inflammatory conditions, microglia are stimulated and induced into an angiogenic phenotype, which exacerbates retinal neovascularization. However, certain subtypes of microglia can help to alleviate neovascularization by phagocytosing neovascular tufts or inducing EC apoptosis. Overall, microglia contribute to both the progression and decay of retinal neovascularization in DR, highlighting the diverse roles of these cells in this disease.

## Targeting microglia to inhibit neovascularization

Neovascularization is a hallmark of PDR, the advanced stage of DR, which can lead to tractional retinal detachment and vitreous hemorrhage, ultimately jeopardizing visual acuity in diabetic patients. Therefore, there is a high demand for developing therapies for PDR based on retinal neovascularization. Panretinal photocoagulation (PRP) is a commonly used surgical intervention to treat DR by destroying new blood vessels using high-energy light. VEGF has been identified as a leading contributor to neovascularization in DR. Anti-VEGF drugs are gaining increasing popularity as non-surgical treatments that can be used independently or in combination with laser photocoagulation [134]. A recent meta-analysis involving five randomized controlled trials revealed more benefits of anti-VEGF over PRP in patients with early PDR [135] but the combination of PRP and anti-VEGF is also widely adapted considering safety profile and cost [136]. Photocoagulation destroys newly formed blood vessels, including surrounding tissue, with immense side effects. Anti-VEGF therapy targets EC proliferation and migration to inhibit pathological angiogenesis. Anti-VEGF therapy has been shown to have limited efficacy and potential side effects, such as increased inflammation and the development of cataracts, while laser photocoagulation is associated with permanent scarring and loss of vision due to damage to healthy retinal tissue [137]. Additionally, both therapies are mainly symptom-focused and do not address the underlying pathophysiology of PDR. An important aspect that has been insufficiently studied so far is the interaction between different cell types. This applies to both DR pathology and the current standard medication with VEGF inhibitors and requires further investigation for sufficient development of therapeutic agents. The complete DR microenvironment, including microglia interactions, is an essential driver for the progression of DR. Therefore, the interactions between cell types are potential treatment targets for DR. Microglia are increased and widely activated in DR patients, and microglia density and activation status influence pathological angiogenesis in both directions pro- and anti-angiogenic [138], indicating that microglia regulation is a potential target for retinal neovascularization inhibition in DR treatment. In this section (Table 2), we discuss treatments that involve microglia regulation to inhibit retinal neovascularization in DR. Besides, the following paragraphs illustrate how the current standard therapies for PDR (using anti-VEGF drugs) affect microglia including the mechanism of action to decrease retinal neovascularization in DR. We will focus on three aspects (1) the decrease of activated microglia, (2) the modulation of microglia polarization, and (3) the inhibition of angiogenic cytokine secretion.

**Table 2:** Treatments targeting microglia to inhibit neovascularization

Mode of action	Drug	Study phase	Clinical trial identifier	Ref.
Decrease of activated microglia	Ranibizumab	Approved		[139]
	Melatonin	Phase II	NCT03478306	[140]
	Melatonin	Phase III	NCT04547439	[140]
	5Z-7-oxozeaenol	Preclinical		[141]
	Cyanidin-3-O-glucoside	Preclinical		[142]
	Magnolol	Preclinical		[143]
	HIF-α inhibitor KC7F2	Preclinical		[144]
Modulation of microglia polarization	Ferulic acid	Preclinical		[122]
	Arginase 1	Preclinical		[53]
Inhibition of angiogenic cytokine secretion	Triamcinolone acetonide	Approved		[145]
	Aldosterone synthase inhibitor FAD286	Preclinical		[146]
	Omega-3-polyunsaturated fatty	Phase III	NCT04499820	[114]
	Omega-3-polyunsaturated fatty	Phase III	NCT04120077	[114]
	Omega-3-polyunsaturated fatty	Phase IV	NCT04140201	[114]
	Glucagon-like peptide-1 receptor agonist NLY0	Preclinical		[101]
	Chlorogenic acid	Preclinical		[147]
	Erianin	Preclinical		[148]
	Celastrol	Preclinical		[149]

### Decrease of activated microglia

An increased number and density of activated microglia are widely observed and associated with pro-angiogenic effects. Couturier et al. used an acute intraocular inflammation model to indicate the influence of anti-VEGF on microglia activation, and they raised a concern that since anti-VEGF drugs are repeatedly injected in the vitreous of patients with retinal diseases, part of their effects could result from unsuspected modulation of the microglia activation state [150]. The decreased hyperreflective foci in DR patients treated with an anti-VEGF drug, Aflibercept, confirmed its effects on the deactivation of retinal microglia [151]. Further, Aflibercept promoted the clearance of neovascular tufts in the OIR retina by modulating microglia activity and increasing the number of ramified resting cells [152]. Another anti-VEGF drug, Ranibizumab, has been also revealed to alleviate the increase in activated microglia in the ischemia retinopathy model [139]. The inhibition of microglia activation is, therefore, a promising tool to decrease neovascularization in DR. Based on this knowledge, it is auspicious to study further substances regarding their microglia resting potential that have not become DR therapeutics, yet. Besides anti-VEGF drugs, some other compounds have been shown to inhibit microglia activation. Initially known for maintaining circadian rhythm, Melatonin has been recently used in retinal vascular proliferative alterations in an experimental model of DR. In OIR model, it was shown to attenuate retinal neovascularization, partly via turning activated microglia back to resting status, with a reduction of almost half in the proportion of amoeboid-activated microglia [140]. Another possibility is the transforming growth factor β activated kinase 1 (TAK-1) inhibitor. TAK-1 expression was upregulated at the transcriptional level in the neovascular region and found to be positively correlated with neovascularization in the OIR model. Inhibition of TAK-1 by 5Z-7-oxozeaenol, a selective TAK-1 inhibitor, attenuated aberrant retinal angiogenesis in the OIR model, with a significant decrease in the number of activated microglia [141]. A further possibility to decrease the number of activated microglia is their depletion, which can be achieved by utilizing certain pharmacological substances, such as diphtheria toxin or CSF1R antagonists [153]. For treatment purposes, depletion of microglia is usually followed by replenishment of microglia from healthy mice donors, which has been found to ameliorate microglia-associated DR pathogenic angiogenesis and vascular damage while providing protective effects for neurons [154, 155]. This approach may offer a promising therapeutic strategy to target microglia in DR while minimizing potential adverse effects.

Except for the already mentioned ones, only a few studies highlighted further drugs altering the number of activated microglia. Three more possible DR drugs may be Cyanidin-3-O-glucoside (C3G), KC7F2 and Magnolol. C3G, a type of anthocyanin, was revealed to alleviate inflammation and microglia activation in DR mice [142]. The HIF1α inhibitor, KC7F2, and Chinese herb medicines, such as Magnolol showed an inhibitory effect on retinal neovascularization via diminished activation of microglia [143, 144].

In conclusion, mounting evidence suggests that the modulation of microglia activation plays a significant role in controlling DR and its associated pathological angiogenesis. Various drugs, including anti-VEGF agents (Aflibercept, Ranibizumab), and other compounds like Melatonin, 5Z-7-oxozeaenol, and Cyanidin-3-O-glucoside, have demonstrated their potential to deactivate or alter activated microglia in DR models, and consequently, suppressed neovascularization of the retina. The emerging understanding of the role of microglia in DR pathogenesis may lead to novel therapeutic strategies that specifically target microglia activation while minimizing potential adverse effects.

### Modulation of microglia polarization

Studies have shown that the polarized M2 phenotype of microglia mitigated pathological angiogenesis. Various drugs aim to promote the polarization from M1 to M2. One of these drugs is Ferulic acid (FA), a plant cell wall compound used in the pharmaceutical, food, and cosmetics industries, but so far not applied as a therapeutic agent for DR in patients [122]. However, it has also been highlighted that a too high proportion of M2 polarized microglia may promote retinal pathological neovascularization instead of being anti-angiogenic [156, 157]. Hence, current studies emphasized therapeutic efficacy for mixed M1 and M2 microglia. Latest research has uncovered the potential therapeutic use of Arginase 1 to modulate microglia polarization [53]. Initially, it has been identified as an essential regulator of the urea cycle in the liver, but was later discovered to be expressed in the retina as well. Intravitreal injection of Arginase 1 showed effectiveness in limiting retinal neovascularization and promoting angiogenic repair by inducing the transition of microglia toward an intermediate and restorative phenotype. This intermediate subtype was positive for both M2-like microglia marker CD206 and M1-like marker CD16/32. Microglia accumulated in areas with elevated formation of tip cells and around the resolving retinal neovascularization [53]. Therefore, a promising and innovative therapeutic approach for managing DR involves modulating microglia polarization and steering them towards the M2 phenotype, while maintaining the appropriate mixture of M1 and M2 microglia. This polarization status holds great potential as a novel therapeutic mechanism. However, further research is needed to determine the optimal combination of M1 and M2 microglia in achieving therapeutic efficacy. Identifying these crucial parameters is essential for developing successful treatment strategies and improving patient outcomes in DR.

### Inhibition of angiogenic cytokine secretion

VEGF is the main angiogenic growth factor involved in DR-related retinal neovascularization. Additionally, various angiogenic factors outside the VEGF family have been reported to be biomarkers for DR [158]. Microglia are the critical source of those angiogenic factors in the retina, e.g., VEGF-A, TNF-α, and IL-1β, which directly or indirectly induce angiogenesis. Thus, inhibiting the secretion of these cytokines could be one strategy to inhibit angiogenesis in DR. To date, several drugs have been revealed to inhibit microglia-secreted angiogenic factors.

Triamcinolone acetonide (TA) is a corticosteroid used in retinal pathologies including PDR for anti-angiogenic and anti-inflammatory effects. Intravitreal injection of dendrimer nanoparticles conjugated to triamcinolone acetonide (D-TA) retained these particles in activated microglia within the retina, resulting in a decrease of IL-1β and TNF-α secretion by 55% and 36%, respectively. Thereby these particles suppressed microglia-induced pathological neovascularization in the OIR model [145]. Furthermore, a considerably reduced level of typical microglia-derived TNF-α and CCL-2 has been detected in the whole retina of an OIR model through the application of an Aldosterone synthase inhibitor, FAD286, leading to reduced neovascularization. Although TNF-α and CCL-2 are known to be released by microglia in retinopathy [159], it still needs to be determined whether other cells contribute to the overall reduction of these cytokines as well [146]. Additionally, omega-3-polyunsaturated fatty acid (ω-3-PUFA) might decrease TNF-α production in a subset of microglia. Hence, an increased intake of ω-3-PUFAs by diet helps to protect against neovascularization. This microglia subgroup that is positive for CSF1R has been typically found in close association with retinal vessels, indicating their potential involvement in retinal angiogenesis [114]. A recent study investigated the impact of ω-3 PUFAs on microglia in vitro further supported this notion, and demonstrated that ω-3 PUFAs suppressed hypoxia-induced retinal neovascularization by inhibiting microglial pyroptosis [160]. Further molecules, such as Glucagon like peptide 1 receptor (GLP1R) agonists have been shown to inhibit microglia secretion of cytokines such as TNF-α and IL-6 [101]. They are in clinical use for glycemic control in diabetes. For PDR, Zhou et al. revealed that NLY0, a glucagon like peptide 1 receptor, suppressed pathologic retinal neovascularization in the OIR mouse model [101].

Moreover, there have been reports on the efficacy of natural products such as chlorogenic acid and Erianin in reducing retinal angiogenesis and attenuating the development of DR. Both drugs have been found to decrease microglia-derived paracrine VEGF-A expression by inhibiting the transcriptional activation of HIF-1α [147, 148]. Another bioactive compound called Celastrol has been identified as a promising compound in alleviating pathological retinal neovascularization in the OIR mouse model. Further, Celastrol suppressed pro-angiogenic cytokines including IL-1, IL-6, TNF-α, and MCP-1, as well as associated enzymes like COX-2 and MMP-9 [149]. These natural products highlight the potential for the identification of new therapeutic agents that target microglia in regulating retinal angiogenesis and may hold promise in the treatment of DR.

In conclusion, regulating microglia through various therapeutic options, such as corticosteroids, dietary interventions (ω-3-PUFA), and receptor agonists (GLP-1R) are promising approaches for inhibiting angiogenesis and attenuating DR progression. Consequently, these therapies appear to work, in part, by regulating the secretion of cytokines from activated microglia or modulating their association with retinal vessels, ultimately suppressing pathological neovascularization in DR. Future analyzes aim to establish the precise mode of action of these compounds. Synergistic effects of these treatments may pave the way for more effective, targeted therapies in managing DR, improving patient outcomes, and preserving vision.

## Conclusions and perspectives

As microglia are essential for vascular development, their role in DR has come under greater scrutiny. Microglia exhibit dual effects on DR: they can aggravate angiogenesis by secreting pro-angiogenic factors, with depletion or inhibition of microglia alleviating neovascularization, but they can also exert protective effects by promoting neovascularization regression through inducing EC apoptosis or physically removing neovascularization tufts. This dual role of microglia highlights the functional heterogeneity of microglia populations within neovascularization lesions.

In DR, microglia activation and migration have been commonly observed before evident vascular changes [91]. Numerous studies have reported early activation and morphological alterations in retinal microglia before retinal neuronal damage and neovascularization, suggesting their potential as biomarkers for early DR detection [161, 162]. Based on these findings, targeting microglia activation, or associated pro-inflammatory molecules may also present a promising approach to diagnosing DR in its early stages.

The modulation of microglia number, activation, and polarization status are three major mechanisms which could inform the development of microglia-targeted therapies for PDR. Partially, current standard therapies using VEGF inhibitors induced these microglia mechanisms. However, several challenges exist in developing effective microglia-based therapies for DR. One of the challenges is to selectively target activated microglia without compromising their protective functions in healthy tissues. Another challenge is to develop effective drug delivery systems that can target microglia specifically in the retina without unwanted influence on microglia in other CNS regions**.**

One of the most pressing research questions in this field is understanding the triggers and regulators of microglial activation in DR. Another critical gap in knowledge is the precise contribution of microglia to the progression of DR. While numerous studies have reported the early activation of microglia in DR, the extent to which this contributes to disease progression versus being a response to retinal injury is not fully understood. This distinction is crucial for developing therapeutic strategies that target microglia without compromising their essential functions in the retina.

In summary, as diabetic retinopathy involves the dysfunction of not only microglia and ECs but also the entire neurovascular unit [11], a comprehensive understanding of microglial functions and interactions within the diabetic retina is crucial for advancing therapeutic strategies for DR. Future research should aim to identify specific microglial subtypes involved in DR in multiple levels in a spatiotemporal context, develop targeted therapies that modulate microglial activity without adverse effects, and explore the role of microglia as biomarkers for early detection and intervention in DR. Addressing these research questions and knowledge gaps will be pivotal in developing effective treatments for this complex and debilitating disease.
